# Seroepidemiological survey of the prevalence of *Helicobacter pylori* infection in Sabah, Malaysia

**DOI:** 10.1016/j.ijregi.2021.12.012

**Published:** 2022-01-05

**Authors:** Daisuke Mori, Jecelyn Leaslie John, Shahnaz Irwani Binti Sabri, Saliz Mazrina Binti Shaharom, Hidekatsu Iha, Yoshio Yamaoka, Takashi Matsumoto, Kamruddin Ahmed

**Affiliations:** a Department of Pathology and Microbiology, Faculty of Medicine and Health Sciences, Universiti Malaysia Sabah, Kota Kinabalu, Sabah, Malaysia; b Borneo Medical and Health Research Centre, Faculty of Medicine and Health Sciences, Universiti Malaysia Sabah, Kota Kinabalu, Sabah, Malaysia; c Department of Transfusion Medicine, Queen Elizabeth Hospital II, Ministry of Health Malaysia, Kota Kinabalu, Sabah, Malaysia; d Department of Microbiology, Faculty of Medicine, Oita University, Oita, Japan; e Department of Environmental and Preventive Medicine, Faculty of Medicine, Oita University, Oita, Japan

**Keywords:** *Helicobacter pylori*, Seroepidemiology, Humans, Sabah, Malaysia

## Abstract

**Objective:**

Gastric cancer is an important cause of mortality in Sabah, Malaysia, but the prevalence of *Helicobacter pylori* infection in this state is unknown. Serology is an important tool for the surveillance of *H. pylori* infection. The objective of this study was to determine the prevalence of *H. pylori* infection by serology, and to provide information for policy discussions on *H. pylori* control programmes in Sabah.

**Methods:**

This cross-sectional study analysed serum samples collected from blood donors in a hospital in Kota Kinabalu, Sabah. *H. pylori* antibody concentration was measured using a commercially available kit.

**Results:**

The prevalence of *H. pylori* was 28.4% (204/718), and the infection rate was highest in subjects aged 30–39 years. Men were more likely to be infected than women. The *H. pylori* infection rate was highest among those of Kadazan (19.1%) ethnicity, followed by Dusun (15.7%), Bajau (14.7%) and Rungus (10.8%). Binary logistic regression analysis indicated that Chinese (*P*=0.026) and Malay (*P*=0.035) ethnicities were protective against *H. pylori* infection compared with other ethnicities.

**Conclusions:**

The seropositivity rate of *H. pylori* in this study was similar to the overall seropositivity rate in Malaysia; however, Chinese and Malay ethnicities were found to be protective against *H. pylori* infection.

## Highlights

• The prevalence of *Helicobacter pylori* was 28.4%, and was highest in subjects aged 30–39 years.
• Men were more likely to be infected than women.
• The *H. pylori* infection rate was highest among those of Kadazan (19.1%) ethnicity.
• Chinese and Malay ethnicities were protective against *H. pylori* infection.

## Introduction

*Helicobacter pylori* is a helically shaped, motile, Gram-negative bacterium (Alzahrani et al., 2014) that was first identified in 1982 and named *Campylobacter pylori* but later classified in the genus *Helicobacter* (Robin Warren and Marshall, 1983; Goodwin et al., 1989). *H. pylori* infects the stomach and can cause ulcers, inflammation of the stomach lining, and cancer (Yamaoka, 2010). Gastric cancer is one of the main causes of cancer-related death worldwide, accounting for 769 million deaths each year (Bray et al., 2018; Rawla and Barsouk, 2019; Sung et al., 2021). People infected with *H. pylori* have a six-fold higher risk of developing gastric cancer (Vohlonen et al., 2016). This infection can be acquired during childhood, and the risk of infection is strongly related to lifestyle factors such as living in crowded conditions, living without good sanitation, and living with infected individuals (Kusters et al., 2006). Infection occurs by direct person-to-person transmission via either the oral–oral or faecal–oral route (Allaker et al., 2002). Although no vaccine against *H. pylori* is currently available, the development of a vaccine is considered to be a powerful tool for preventing gastric cancer (Sutton and Boag, 2019).

In Malaysia, studies have shown a seropositive rate for *H. pylori* of 14.1–55% among blood donors (Uyub et al., 1994; Goh and Parasakthi, 2001; Sasidharan and Uyub, 2009). In studies of ethnic groups within Malaysia, the infection rate was reported to be higher in people of Chinese and Indian ethnicities than in those of Malay ethnicity (Goh and Parasakthi, 2001; Kaur and Naing, 2003; Alfizah et al., 2010). Most of these studies were performed in west Malaysia, and few studies have been undertaken in Sabah, a state with various ethnic groups that showed a high seroprevalence rate of 55% in a study conducted 16 years ago (Goh and Parasakthi, 2001). Although the seroprevalence of *H. pylori* is closely related to the incidence of gastric cancer (Rahman et al., 2014), the incidence of gastric cancer is low (range 4.3–5.9%) (Kaur et al., 2016, Suleiman et al., 2015). This discrepancy suggests that further studies are needed to determine the prevalence of *H. pylori* among the people of Sabah. However, testing for *H. pylori* by tissue biopsy and culture is impractical for a large number of people, and analysis using serology may be a more practical method to determine the prevalence of *H. pylori* among the population. Therefore, this surveillance study was conducted to determine the prevalence of *H. pylori* and to provide data for policy discussions on *H. pylori* control programmes in Sabah.

## Materials and methods

### The study

From August 2016 to April 2019, blood samples were collected from blood donors who attended the Department of Blood Transfusion for blood donation at Hospital Queen Elizabeth II, Kota Kinabalu, Sabah, Malaysia. Physically and mentally healthy blood donors aged ≥18 years were enrolled in this study. Selection of participants was also based on the distribution of the major ethnic groups in Sabah. For each ethnic group, when ≥100 samples were collected, the first 100 samples were analysed, but when ≤100 samples were collected, all samples were analysed for antibodies against *H. pylori.*

Demographic data including gender, age and ethnicity were recorded as the possible independent predictors of, or protective factors against, infection. Three millilitres of blood were collected from each participant, left at room temperature for clot formation, and centrifuged at 3000 rpm for 15 min to separate the serum. The serum was transferred to a cryotube using sterile procedures, and serum samples were transported via cold chain to the laboratory of Borneo Medical and Health Research Centre, Universiti Malaysia Sabah. Samples were stored at –80°C until further analysis.

The concentration of serum antibodies against *H. pylori* was measured with a solid-phase two-step sandwich enzyme immunoassay using a commercial kit (E-Plate Eiken *H. pylori* antibody II, Eiken Chemical Co., Ltd., Tokyo, Japan) according to the manufacturer's instructions. The cut-off value of 10 U/mL was considered to be a positive indicator of antibodies against *H. pylori.* The sensitivity and specificity of this kit are 91.2% and 97.4%, respectively (Ueda et al., 2014).

Statistical analysis was performed using SPSS Version 27 (IBM Corp., Armonk, NY, USA). All categorical variables are described as frequency and percentage. Chi-squared test was used to investigate the associations of *H. pylori* infection with gender, age group and ethnicity. Variables with *P*<0.05 in the univariate analyses were selected for multi-variate analysis using binary logistic regression to examine their relationship with *H. pylori* infection. Odds ratios (ORs) and 95% confidence intervals (CIs) were calculated, and *P*-values <0.05 were considered to indicate significance.

### Ethical approval

The authors assert that all procedures contributing to this work comply with the ethical standards of the relevant national and institutional committees on human experimentation and with the Helsinki Declaration of 1975, as revised in 2008 and approved by Medical Research and Ethics Committee, Ministry of Health Malaysia (NMRR-19-699-47195).

## Results

Serum samples were collected from 718 blood donors. Their mean age was 34.8 years (range 18–66 years). The male-to-female ratio was 3.4:1, with 556 (77.4%) men and 162 (22.6%) women. Blood donors were from 14 ethnic groups; most were Kadazan, followed by Dusun, Bajau, Malay and Chinese.

The prevalence of serum antibodies against *H. pylori* was 28.4% (204/718). The male-to-female ratio of seropositive samples was 3.4:1, with 158 (77.5%) and 46 (22.5%) positive samples from men and women, respectively. The *H. pylori* infection rate was highest in people of Kadazan (19.1%, 39/204) ethnicity, followed by Dusun (15.7%, 32/204), Bajau (14.7%, 30/204) and Rungus (10.8%, 22/204). The sample was classified according to six age group categories: 18–19, 20–29, 30–39, 40–49, 50–59 and 60–69 years. The highest infection rate was in subjects aged 30–39 years. The prevalence of *H. pylori* seropositivity differed significantly between ethnic groups (*P*=0.000), but did not differ between men and women (*P*=0.825) or between age groups (*P*=0.936) (Table 1). The univariate analysis showed that people of Chinese (*P*=0.000), Kadazan (*P*=0.011), Malay (*P*=0.001) and Rungus (*P*=0.000) ethnicities had higher seroprevalence for *H. pylori* than other ethnic groups. Binary logistic regression analysis (Table 2) showed that being Chinese (*P*=0.026) and Malay (*P*=0.035) were significant protective factors against having *H. pylori* infection compared with other ethnic groups.

**Table 1:** Demographic characteristics and univariate analysis of *Helicobacter pylori* infection among blood donors (*n*=718).

	Total subjects *n* (%)	Positive *n* (%)	OR (95% CI)	*P*-value
Gender				
Male	556 (77.4)	158 (77.5)	0.96 (0.65–1.41)	0.825
Female	162 (22.6)	46 (22.5)	Reference	
Age group (years)				
18–19	10 (1.4)	2 (1.0)	0.63 (0.13–2.97)	0.553
20–29	234 (32.6)	63 (30.9)	0.90 (0.63–1.27)	0.538
30–39	243 (33.8)	68 (33.3)	0.97 (0.69–1.37)	0.855
40–49	141 (19.6)	44 (21.6)	1.18 (0.79–1.76)	0.412
50–59	83 (11.6)	25 (12.3)	1.10 (0.67–1.81)	0.714
60–69	7 (1.0)	2 (1.0)	Reference	
Ethnicity				
Bajau	100 (13.9)	30 (14.7)	1.09 (0.69–1.74)	0.704
Bisaya	13 (1.8)	2 (1.0)	0.45 (0.10–2.06)	0.293
Brunei	71 (10)	25 (12.3)	1.42 (0.85–2.38)	0.181
Bugis	15 (2.1)	4 (2.0)	0.91 (0.29–2.91)	0.880
Chinese	100 (13.9)	13 (6.4)	0.33 (0.18–0.61)	**0.000**
Dusun	100 (13.9)	32 (15.7)	1.22 (0.77–1.92)	0.391
Iban	9 (1.3)	2 (1.0)	0.72 (0.15–3.48)	0.679
Indian	10 (1.4)	2 (1.0)	0.63 (0.13–2.97)	0.553
Kadazan	100 (13.9)	39 (19.1)	1.76 (1.13–2.72)	**0.011**
Malay	100 (13.9)	14 (6.9)	0.37 (0.20–0.66)	**0.001**
Murut	19 (2.6)	9 (4.4)	2.33 (0.93–5.81)	0.063
Rungus	42 (5.8)	22 (10.8)	2.99 (1.59–5.60)	**0.000**
Sino Dusun/Kadazan	26 (3.6)	5 (2.5)	0.59 (0.22–1.59)	0.290
Sungai	13 (1.8)	5 (2.5)	Reference	

OR, odds ratio; CI, confidence interval.

**Table 2:** Binary logistic regression of *Helicobacter pylori* seropositivity according to ethnicity (*n*=718).

	Adjusted OR	95% CI	*P*-value
Ethnicity			
Chinese	0.24	0.07–0.84	0.026
Malay	0.26	0.07–0.91	0.035

OR, odds ratio; CI, confidence interval.

## Discussion

Globally, there is a declining trend in the seroprevalence of *H. pylori*, and Malaysia is reflecting this trend (Hooi et al., 2017; Hee et al., 2018; Hanafiah et al., 2019). In agreement with this trend, the *H. pylori* seropositivity rate (28.4%) in the present study was considerably lower than the rate of 55.0% reported 16 years ago (Goh and Parasakthi, 2001). The rate in the current study is also similar to the rate of 28.6% for Malaysia as a whole (Hooi et al., 2017). However, the seroprevalence of *H. pylori* is lower in neighbouring Indonesia (14.3–22.1%) (Syam et al., 2015). In contrast, the seroprevalence of *H. pylori* is higher in neighbouring Thailand and the Philippines (54.1–76.1% and 60%, respectively) (Destura et al., 2004; Sahara et al., 2012). The reasons behind the declining seroprevalence rate in Sabah are unknown but may reflect improvements in the sanitation system (Tan and Goh, 2008; Watanabe et al., 2015). Differences between the test kits used in these studies may also affect the detection of seroprevalence (Vaira et al., 1994). However, the lower prevalence of *H. pylori* found in this study may explain the low prevalence of gastric cancer in Sabah (Kaur et al., 2016).

Similar to a trend reported previously (Goh and Parasakthi, 2001), the present study also found that men were more likely to be seropositive for *H. pylori* than women. However, other studies have not found a gender difference in seroprevalence (De Martel and Parsonnet, 2006; Franck et al., 2017; Bálint et al., 2019), which may reflect genetic variations between people living in different geographic regions. Also in agreement with a previous study (Goh and Parasakthi, 2001), the present study found that people aged 20–39 years were at higher risk of testing positive for *H. pylori* compared with those aged ≥40 years. Another study reported that subjects aged 41–60 years had significantly lower prevalence because of declining *H. pylori* strains that carry the CagA virulence factor (Franck et al., 2017).

According to the racial cohort theory proposed in other studies, the rate of *H. pylori* infection is higher in people of Chinese and Indian ethnicities than in those of Malay ethnicity (Kang et al., 1990; Goh, 1997; Goh and Parasakthi, 2001). As in the present study, Goh and Parasakthi (2001) found that the rate of infection was highest in people of Kadazan ethnicity in Malaysia. However, the binary logistic multi-variate analysis in the present study showed that Chinese and Malay ethnicities were protective against *H. pylori* infection, and that seroprevalence was low and similar in both groups. Other studies have reported that ethnicity is a major significant risk factor for *H. pylori* infection in regions where the levels of *H. pylori* strains with different CagA virulence factors differ between ethnic groups ( Den Hollander et al., 2013, Goh, 1997). The presence of different virulence factors of *H. pylori* can also influence the outcome of infection in different ethnic groups (Hanafiah and Lopes, 2020). More studies are needed to identify the virulence factors carried by *H. pylori* colonizing people of Chinese and Malay ethnicities in Sabah. It is possible that the similarity in host genetic factors between people of Chinese and Malay ethnicities may be related to their similar susceptibility to *H. pylori* infection (Tan and Goh, 2008).

Although there is no universally accepted single test for the diagnosis of *H. pylori* infection, culture and histology results are considered as the gold standards (Rojas-Rengifo et al., 2019). Studies showed that when culture was taken as the gold standard, the sensitivity and specificity of serological tests were 80–95% and 80 – 95%, respectively (Logan and Delaney, 2001). When histology was considered as the gold standard, the sensitivity and specificity of serological tests were 76–84% and 79–90%, respectively (Chey and Wong, 2007). Furthermore, the sensitivity, specificity, and positive and negative predictive values of different commercially available serological tests vary considerably (Burucoa et al., 2013). Despite these limitations, serological testing is a convenient method for mass screening for *H. pylori* infection. In the present study, there was a possibility of misidentification of *H. pylori* infection due to the use of serological testing.

The major limitations of this study are that most of the blood donors were men and, because of limited resources, only three variables were included in this study. Other factors, such as diet, genetic predisposition, environmental and socio-economic conditions, and cultural background, are crucial factors that can affect susceptibility to *H. pylori* infection, and should be included in future studies. In addition, future studies could enrol family members to examine the role of intrafamily transmission, which has been reported to be important to the spread of *H. pylori*. Despite these limitations, this cross-sectional study provides updated data on the seroprevalence of *H. pylori* infection among the population of Sabah. These data can be used to support regional initiatives for *H. pylori* surveillance, prevention and eradication programmes.

In conclusion, this study found a relatively low seroprevalence rate for *H. pylori* among the population of Sabah, and that people aged <40 years were mainly affected. Chinese and Malay ethnicities seem to be protective against the development of *H. pylori* infection in this population.
